# Assessment of the association between health problems and cooking fuel type, and barriers towards clean cooking among rural household people in Bangladesh

**DOI:** 10.1186/s12889-024-17971-7

**Published:** 2024-02-19

**Authors:** Sayed Mohammad Rasel, Abu Bakkar Siddique, Md. Fahad Shahariar Nayon, Md Shakil Mahmud Suzon, Sanzida Amin, Sadia Sultana Mim, Md. Shakhaoat Hossain

**Affiliations:** 1 Department of Public Health and Informatics, Jahangirnagar University, Savar, Dhaka, 1342 Bangladesh; 2 Air Pollution, Climate Change and Health (ACH) Lab, Department of Public Health and Informatics, Jahangirnagar University, Savar, Dhaka, 1342 Bangladesh; 3 Centre for Advanced Research Excellence in Public Health, Savar, Dhaka, 1342 Bangladesh; 4 Department of Public Health, School of Pharmacy and Public Health, Independent University, Bangladesh (IUB), Dhaka, Bangladesh

**Keywords:** Cooking fuel type, Clean cooking, Health problems, Solid fuel, Barriers, Bangladesh

## Abstract

**Background:**

In low- and middle-income countries, households mainly use solid fuels like wood, charcoal, dung, agricultural residues, and coal for cooking. This poses significant public health concerns due to the emission of harmful particles and gases. To address these issues and support Sustainable Development Goals (SDGs), adopting cleaner cooking fuels like electricity and gas are acknowledged as a viable solution. However, access to these cleaner fuels is limited, especially in rural areas.

**Methods:**

This study conducted a face-to-face survey with 1240 individuals in rural Bangladesh to explore the link between health issues and cooking fuel type, as well as barriers to transitioning to clean cooking. Using a convenient sampling technique across four divisions/regions, the survey gathered socio-demographic and health data, along with information on clean cooking barriers through a semi-structured questionnaire. Binary and multivariable logistic regression analyses were then employed to identify significant associations between cooking fuel type and health problems.

**Results:**

The study revealed that a majority of participants (73.3%) relied on solid fuel for cooking. The use of solid fuel was significantly correlated with factors such as lower education levels, reduced family income, location of residence, and the experience of health issues such as cough, chest pressure while breathing, eye discomfort, diabetes, asthma, and allergies. Economic challenges emerged as the foremost obstacle to the adoption of clean cooking, accompanied by other contributing factors.

**Conclusion:**

The use of solid fuel in rural Bangladeshi households poses substantial health risks, correlating with respiratory, eye, cardiovascular, and metabolic issues. Lower education and income levels, along with specific residential locations, were associated with higher solid fuel usage. Economic challenges emerged as the primary obstacle to adopting clean cooking practices. These findings emphasize the need for implementing strategies to promote clean cooking, address barriers, and contribute to achieving Sustainable Development Goal targets for health and sustainable energy access in Bangladesh.

## Introduction

In low- and middle-income countries, household cooking primarily relies on solid fuels such as wood, charcoal, dung, agricultural residues, and coal [1]. However, their use has raised significant public health concerns and garnered considerable attention. Traditional open-fire cooking methods are highly inefficient and release harmful particulates and gaseous substances into the household air, causing approximately 4 million premature deaths annually [2], with women and children being particularly vulnerable [3]. Literature indicates that the combustion of solid fuels in inefficient stoves produces hazardous substances, including suspended particulate matter (PM), carbon monoxide, poly-aromatic hydrocarbons, poly-organic matter, and formaldehyde, resulting in severe health consequences [4]. These adverse effects are associated with public health hazards such as reduced lung function, respiratory illnesses, asthma, pneumonia, tuberculosis, eye disorders, pregnancy complications, cardiovascular disease, and cancer [5]. Furthermore, traditional domestic energy methods also have detrimental effects on health, safety, and overall well-being [6].

Compared to solid fuels, cleaner alternatives like electricity and gases are often considered healthier and less detrimental to human health [7]. Cooking with clean fuel supports women’s empowerment, provides public health benefits, and reduces health damage from indoor air pollution [8]. Recognizing the extensive negative consequences of polluting solid cooking fuels, one of the Sustainable Development Goals (SDGs) prioritizes universal access to clean cooking (part of SDG target 7.1) [9]. This target has been directly linked to achieving various other SDG targets as well [10]. While clean cooking fuels or gases are prevalent in cities, biomass fuels continue to be widely used in rural areas. Current projections suggest that a third of the world’s population will still rely on polluting fuels in 2030 [2]. Efforts have been made over time to elucidate the factors contributing to this outcome.

Bangladesh, a lower-middle-income country, has the lowest access to clean cooking fuels. Approximately 94% of rural Bangladeshi households cook and heat using solid fuels like wood, coal, and animal dung [11]. Previous studies in Bangladesh have demonstrated the negative association of cooking with solid fuels with respiratory and eye health [12], cardiovascular health [13], under-five mortality [14], and adverse obstetric outcomes among women exposed to biomass fuel [15]. Solid fuel users, in comparison to clean fuel users, exhibited significantly higher blood pressure [16]. Furthermore, household air pollution (HAP) from solid fuel cooking has emerged as a significant contributor to fatalities and disabilities in many developing countries, including Bangladesh [17].

There exists a significant barrier or gap hindering the widespread acceptance and usage of clean cooking fuels in Bangladesh and globally, which poses a notable challenge [18]. Researchers attribute this barrier to a range of factors, including socio-cultural, economic, political, and institutional challenges [19]. Without addressing and dismantling these barriers through the implementation of integrated economic and social policies, the realization of various Sustainable Development Goal (SDG) targets remains an aspirational goal with limited chances of success [20].

This study aims to contribute to the existing literature by highlighting the comparison between health issues and cooking fuel categories, along with barriers to adopting clean cooking. The fuel categories are broadly classified into two groups. Initially, we assessed the predominant fuel types used by participants, categorizing them into: solid fuel and clean fuel, in alignment with findings from prior research [21, 22]. Given that the outcome or dependent variable in our study is binary, we opted for binary logistic regression, consistent with established practices outlined in the existing literature [23, 24]. The study investigates and compares health problems associated with these cooking fuels, specifically analyzing the frequency of health issues experienced by rural individuals when using solid fuels compared to clean fuels. Subsequently, the research delves into identifying barriers towards clean cooking in rural settings, despite the numerous health benefits associated with it.

Our study introduces two novel aspects. Firstly, unlike previous studies that primarily concentrate on the health effects of solid fuels within limited regions, we compare the health effects across different fuel categories, encompassing a large geographical area in rural Bangladesh with a substantial sample size. Secondly, despite some global studies addressing clean cooking barriers, none specifically focus on these barriers in Bangladesh. Our study offers valuable insights into the challenges of adopting clean cooking practices in rural Bangladesh. The transition to clean energy sources is a topic under consideration by regional governments, yet little has been explored regarding the potential health effects of such a shift. Our findings can provide valuable information for policymakers dealing with this specific population.

## Literature review

### Association between health problems and cooking fuel type

Household cooking fuel usage is associated with various health issues, with research indicating that opting for solid fuel over clean fuel negatively affects health [25]. A significant portion of the population, especially in low- and middle-income countries, lacks access to clean cooking facilities and predominantly uses solid biomass fuel for cooking [1]. Traditional solid-fuel cooking produces greenhouse gases and harmful air pollutants, increasing the risk of respiratory disease, cardiovascular disease, cerebrovascular disease, blindness, and neonatal mortality [26]. Women and children under 5 are particularly vulnerable to these health threats due to their increased involvement in cooking-related activities [3].

In Bangladesh, where this study was conducted, a substantial portion of the population relies on solid fuels such as coal, lignite, charcoal, and wood for cooking [27]. Various regional studies in Bangladesh have highlighted the association between this practice and adverse health outcomes, including respiratory symptoms [28], cardiovascular morbidity and mortality [29], eye problems [30], adverse pregnancy outcomes [17], elevated blood pressure [13], under-5 mortality [14], and respiratory diseases [31]. Furthermore, a study investigated that household air pollution (HAP) from cooking with solid fuels has become a leading cause of death and disability in many developing countries, including Bangladesh [17].

Most studies in Bangladesh have focused on the health effects of solid fuels within specific regions rather than comparing them with clean cooking fuels. This study aims to compare health problems between clean and solid fuels, considering a relatively large geographical area to provide a comprehensive perspective on the issue.

### Key barriers towards clean cooking

In recent decades, various global organizations, international development agencies, regional governments, and the private sector have endeavored to promote the widespread use and adoption of cleaner cooking methods [32]. Numerous studies highlight the positive impacts of clean cooking, encompassing health and environmental benefits, as well as the value of time saved from collecting fuelwood, which counterbalances the costs associated with implementing clean cooking. Governments and non-governmental organizations in many developing countries, with the support of multilateral and bilateral funding organizations, have implemented several initiatives and programs over the past four decades to encourage clean cooking. However, the acceptance of clean cooking has been exceptionally slow [33], attributed to interconnected, deeply rooted socioeconomic, cultural, and technological factors. Economic and cultural barriers significantly contribute to the slow adoption of clean cooking [34].

A literature review was conducted to identify market barriers to the transition to clean cooking in Sub-Saharan Africa. One major hindrance to the use of clean cooking fuel is the cost to the consumer, which can be substantially higher in numerous places than the cost of conventional fuel. The transition to clean cooking fuels is complicated by a lack of distribution infrastructure and insufficient information exchange between producers, consumers, and intermediary organizations. In addition to the factors mentioned above—being the most significant barriers to fuel switching—there are various minor social and cultural issues that reinforce reliance on solid fuel [35].

Several systematic literature reviews on clean cooking from the demand-side perspective have already been published. For instance, one study analyzed 32 papers to identify the determinants of fuel and stove choice [36], while another review examined 44 studies focusing on the adoption of clean fuels [37]. Bonan et al. [38] conducted a review to identify the barriers to and drivers of the adoption of different types of clean fuels and their impact on economic development and poverty reduction.

While there is a substantial body of literature on clean cooking barriers globally, there is a notable absence of specific research on this topic in Bangladesh. Only a limited number of studies have attempted to address this issue, but they lack clear definition, organization, or appropriateness on the matter [39–41]. Despite the significance of these contributions, there is a pressing need for a more comprehensive analysis of the factors influencing the adoption of clean cooking from a consumer perspective in rural Bangladesh. This study offers valuable insights into the specific barriers to clean cooking and provides guidance for policymakers to initiate potential measures for the adoption of clean cooking practices.

## Methods

### Research framework

**Fig. 1:**
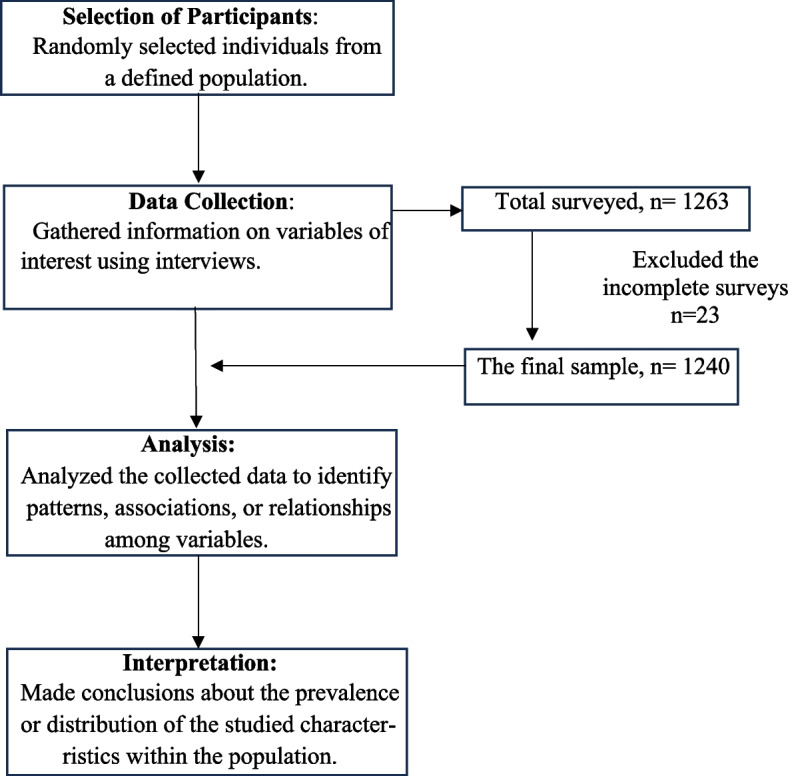
Research framework

Research Framework of this study are demonstrated in Fig. 1.

### Participants and procedures

Between January and May 2023, a cross-sectional face-to-face survey was conducted, involving 1240 rural individuals. The sample was selected using a convenient sampling technique. A semi-structured questionnaire, accompanied by informed consent, was employed to gather information during the face-to-face interviews. Participants had to be 18 years or older, reside in a rural area, and be directly or indirectly involved in cooking to qualify. Those under the age of 18 and individuals who declined to consent were excluded. Following the acquisition of informed consent, 1263 participants were surveyed. Subsequently, incomplete surveys were excluded, and data from 1240 participants (83.7% female; mean age 37.59 years [SD = 11.34]) were included in the analyses.

### Sample size determination

The sample size was calculated using the following equation:

$$n=\frac{{z}^{2}pq}{{d}^{2}};n=\frac{{1.96}^{2}\times 0.5\times \left(1-0.5\right)}{{0.05}^{2}}=384.16\approx 384$$

Here,

*n* = number of samples.

*z* = 1.96 (95% confidence level).

*p* = prevalence estimate (50% or 0.5).

*q* = (1-*p*).

*d* = Precession of the prevalence estimate (10% of 0.05).

As there is no prior study concentrating on association between health problems and cooking fuel type, and barriers to clean cooking in Bangladesh simultaneously, we determined that the best assumption (p) for the current study would be 50%. With a 10% non-response rate, a sample size of 423.5 ≈ 424 participants was predicted. The size of our sample exceeded this projection.

### Study area

**Fig. 2:**
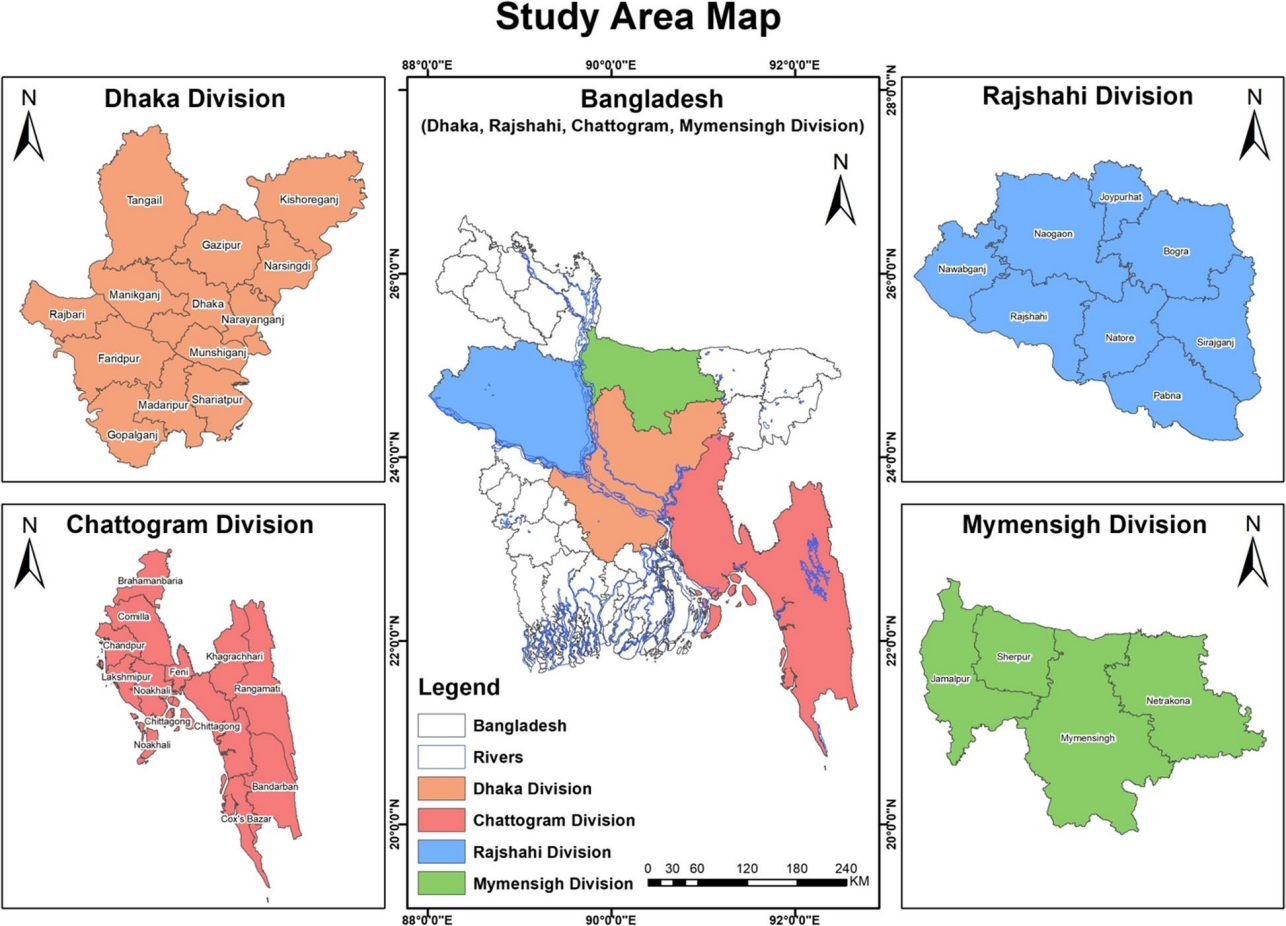
Map of the study areas

The study areas consist of four divisions namely Dhaka, Chattogram, Rajshahi and Mymensingh in Bangladesh (Fig. 2). The data was collected from rural areas of these divisions conveniently.

### Measures

A semi-structured questionnaire full of informed consent along with three sections (i.e., socio-demographics, health related information and information about barriers towards clean cooking) was utilized during data collection.

#### Socio-demographic information

During the survey, some socio-demographic questions were asked including age (later categorized as 18–29 years/ 30–39 years/ ≥ 40 years) [42]; sex (male/female), educational status (Illiterate/primary/secondary/higher secondary/university or above), marital status (married/unmarried/divorced) [43], family type (nuclear [two parents and their children]/joint [family unit with more than two parents] [44];), monthly family income (later categorized: lower socio-economic status (SES) < 15,000 Bangladeshi Taka [BDT], middle SES 15,000–30,000 BDT and upper SES > 30,000 BDT) [45], divisions Dhaka/Chattogram/Rajshahi/Mymensingh) and duration of living (< 2 years/2–5 years/ > 5 years).

#### Dependent variable

During the survey, a question was asked about cooking fuel type (e.g., Which type of fuel do you primarily or mainly use for cooking? Solid fuel *(wood, charcoal, dung, agricultural residues, coal *etc*./* Clean fuel* (liquified petroleum gas (LPG), electricity, and piped gas *etc*.)* [21].

#### Health related information

Throughout the survey, participants were queried about various health-related conditions, including hypertension, allergies, asthma, and diabetes. Responses to these conditions involved three options: ‘Yes’ indicating the clinical presence of the disease, ‘No’ indicating its clinical absence, and ‘Don’t know’ indicating either an absence of diagnostic attempts or lack of awareness about the presence or absence of the disease [21]. Additionally, participants were asked about other health effects, such as sneezing, coughing, breathing difficulties, chest pressure while breathing, itching or burning in the eyes, redness in the eyes, and eye discomfort, with two possible responses: ‘Yes’ or ‘No’.

#### Information about barriers towards clean cooking

While collecting data, participants were asked a question about barriers to clean cooking. The possible barriers included economic problems, attitudes towards technology, problems related to clean fuel distribution, lack of information about clean fuel, a shortage of clean fuel or its unavailability, socio-cultural norms, customs, practices, maintenance issues with clean fuel, a tendency to lose taste in food, social factors, and a lack of awareness of the risks associated with unclean fuel. Multiple responses were allowed for this question.

### Statistical analysis

The data analysis was carried out using Microsoft Excel 2019 and SPSS version 25.0 (Chicago, IL, USA). Microsoft Excel was used to clean, modify, sort, and code the data. The spreadsheet was then imported into the SPSS software. Descriptive statistics (frequency, percentages, means, and standard deviations) as well as first-order analysis (chi-square tests, Fisher’s exact tests) were used. Finally, binary and multivariable logistic regression models were used to determine the relationship between solid fuel use and socio-demographic information. All statistical tests having a *p*-value less than 0.05 were considered significant at 95% confidence interval.

## Results

The socio-demographic characteristics of the participants are introduced in Table 1. A total number of 1240 participants were included in this study, the majority of whom were female (83.4%) and fell within the age range of ≥ 40 years (42%). The majority of the participants (75.7%) were housewife where largest proportion of the respondent from lower socioeconomic status (59.8%). Additionally, Majority of respondents (41.8%) were from Chattogram division followed by Dhaka (28.3%), Rajshahi (16.9%), Mymensingh (13%) and largest proportion of people are living there over 5 years (85.9%). Solid fuels are used for cooking in majority households (73.3%) compared to clean fuel (26.7%). In terms of health-related information, a notable number of respondents had respiratory-related symptoms (cough 11.1%, Pressure in chest while breathing 11% etc.), eye related problems (Itching or burning in eyes 16.9%, Discomfort in eyes 17.9% etc.), diabetes (12.4%), hypertension (21.1%), and allergy (32.6%).

**Table 1:** Socio-demographic characteristics of the participants (*N* = 1240)

**Variables**	n (%)
**Age**	
18–29 years	297 (24%)
30–39 years	422 (34%)
≥ 40 years	521 (42%)
**Gender**	
Male	202 (16.3%)
Female	1038 (83.7%)
**Education**	
Illiterate	161 (13%)
Primary	453 (36.5%)
Secondary	430 (34.7%)
Higher secondary	117 (9.4%)
University or above	79 (6.4%)
**Marital status**	
Married	1133 (91.4%)
Unmarried	74 (6%)
Divorced	33 (2.7%)
**Family type**	
Nuclear family	882 (71.1%)
Joint family	358 (28.9%)
**Monthly family Income**	
Under 15000 BDT (lower SES)	742 (59.8%)
15000 BDT-30000BDT (middle SES)	335 (27%)
More than 30000BDT (higher SES)	163 (13.1%)
**Occupation**	
Student	42 (3.4%)
Housewife	939 (75.7%)
Employee	161 (13%)
Others	98 (7.9%)
**Divisions/Regions**	
Dhaka	351 (28.3%)
Chattogram	518 (41.8%)
Rajshahi	210 (16.9%)
Mymensingh	161 (13%)
**Duration of living**	
< 2 years	23 (1.9%)
2–5 years	152 (12.3%)
> 5 years	1065 (85.9%)
**Fuel type (Dependent/outcome variable)**	
Clean fuel	331 (26.7%)
Solid fuel	909 (73.3%)
**Health related information**	
**Sneeze**	
Yes	72 (5.8%)
No	1168 (94.2%)
**Cough**	
Yes	138 (11.1%)
No	1102 (88.9%)
**Problems in breathing**	
Yes	76 (6.1%)
No	1164 (93.9%)
**Pressure in chest while breathing**	
Yes	137 (11%)
No	1103 (89%)
**Itching or burning in eyes**	
Yes	210 (16.9%)
No	1030 (83.1%)
**Redness in eyes**	
Yes	116 (9.4%)
No	1124 (90.6%)
**Discomfort in eyes**	
Yes	222 (17.9%)
No	1018 (82.1%)
**Diabetes**	
Yes	154 (12.4%)
No	842 (67.9%)
Don’t know	244 (19.7%)
**Hypertension**	
Yes	262 (21.1%)
No	698 (56.3%)
Don’t Know	280 (22.6%)
**Asthma**	
Yes	91 (7.3%)
No	1023 (82.5%)
Don’t know	126 (10.2%)
**Allergy**	
Yes	404 (32.6%)
No	691 (55.7%)
Don’t know	145 (11.7%)

*BDT* Bangladeshi Taka, 1 *BDT equal to* 0.0091 U$$ in 23 September, 2023, *SES* Socio-economic status

Table 2 shows identified factors associated with cooking fuel type. The adjusted models contained the variables shown to be significant in the binary logistic regression analysis. In the adjusted model, participants who had no educational qualification, primary and secondary level education were 8.53 times, 7.60 times and 4.69 times more likely to have solid fuel user (AOR [Adjusted odds ratio] = 8.53, 95% CI [Confidence interval] = 3.22 – 22.61, *p* < 0.001, AOR = 7.60, 95% CI = 3.33 – 17.34, *p* < 0.001 and AOR = 4.69, 95% CI = 2.19 – 10.05, *p* < 0.001, respectively) compared to participants who had studied university or above. Participants from lower socioeconomic status were 13.27 times and 5.25 times more likely to solid fuel use (AOR = 13.27, 95% CI = 6.82 – 25.85, *p* < 0.001 and AOR = 5.25, 95% CI = 2.87 – 9.60, *p* < 0.001, respectively) compared to participants who had higher socioeconomic status. Respondents who lived in Chattogram and Rajshahi division were 6% and 10% less likely to have solid fuel user (AOR = 0.06, 95% CI = 0.04–0.12, *p* < 0.001 and AOR = 0.10, 95% CI = 0.05–0.20, *p* < 0.001, respectively) compared to respondents who lived in Dhaka division. People who were living for 2 to 5 years in their location were 25% less likely to have solid fuel use compared to people who were living for less than 2 years (AOR = 0.25, 95% CI = 0.07 – 0.84, *p* = 0.024).

**Table 2:** Binary and multivariate logistic regression analysis of factors associated with fuel type

**Variables**	**Clean fuel** n (%)	**Solid fuel** n (%)	**Unadjusted model**		**Adjusted model**	
			COR (95% CI)	*p*-value	AOR (95% CI)	*p*-value
**Age**						
18–29 years	108 (8.8%)	188(15.2%)	Reference		Reference	
30–39 years	97 (7.8%)	325 26.2%)	1.94 (1.40–2.70)	** < 0.001**	0.84 (0.52–1.36)	0.470
≥ 40 years	125 (10.1%)	396 (31.9%)	1.84 (1.35–2.50)	** < 0.001**	0.60 (0.34–1.04)	0.070
**Gender**						
Male	54 (4.4%)	148 (11.9%)	Reference		--------------------	
Female	277 (22.3%)	761 (61.4%)	1.00 (0.71–1.41)	0.989		
**Education**						
Illiterate	25 (2.0%)	136 (11.0%)	13.25 (6.94–25.27)	** < 0.001**	8.53 (3.22–22.61)	** < 0.001**
Primary	68 (5.5%)	385 (31.0%)	13.79 (7.96–23.88)	** < 0.001**	7.60 (3.33–17.34)	** < 0.001**
Secondary	119 (9.6%)	311 (25.1%)	6.36 (3.75–10.84)	** < 0.001**	4.69 (2.19–10.05)	** < 0.001**
Higher secondary	63 (5.1%)	54 (4.4%)	2.09 (1.14–3.83)	** < 0.017**	1.42 (0.65–3.14)	0.382
University or above	56 (4.5%)	23 (1.9%)	Reference		Reference	
**Marital status**						
Married	280 (22.6%)	853 (68.8%)	2.54 (1.26–5.01)	**0.009**	2.17 (0.86–5.44)	0.099
Unmarried	36 (2.9%)	38 (3.1%)	0.88 (0.39–2.00)	0.760	1.21 (0.36–4.13)	0.760
Divorced	15 (1.2%)	18 (1.5%)	Reference		Reference	
**Family type**						
Nuclear family	218 (17.6%)	664 (53.5%)	1.41 (1.07–1.84)	**0.014**	0.84 (0.57–1.23)	0.367
Joint family	113 (9.1%)	245 (19.8%)	Reference		Reference	
**Monthly family Income**						
Lower SES	120 (9.7%)	622 (50.2%)	6.39 (4.44–9.21)	** < 0.001**	13.27(6.82–25.85)	** < 0.001**
Middle SES	121 (9.8%)	214 (17.3%)	2.18 (1.49–3.19)	** < 0.001**	5.25 (2.87–9.60)	** < 0.001**
Higher SES	90 (7.3%)	73 (5.9%)	Reference		Reference	
**Occupation**						
Student	20 (1.6%)	22 (1.8%)	Reference		Reference	
Housewife	230 (18.5%)	709 (57.2%)	2.80 (1.50–5.23)	**0.001**	0.90 (0.31–2.62)	0.853
Employee	74 (6.0%)	87 (7.0%)	1.07 (0.54–2.11)	0.848	0.74 (0.24–2.21)	0.584
Others	7 (0.6%)	91 (7.3%)	11.82 (4.44–31.45)	** < 0.001**	2.90 (0.78–10.73)	0.112
**Divisions/Regions**						
Dhaka	39 (3.1%)	312 (25.2%)	Reference		Reference	
Chattogram	202 (16.3%)	316 (25.5%)	0.20 (0.13–0.29)	** < 0.001**	0.06 (0.04–0.12)	** < 0.001**
Rajshahi	77 (6.2%)	133 (10.7%)	0.22 (0.14–0.33)	** < 0.001**	0.10 (0.05–0.20)	** < 0.001**
Mymensingh	13 (1.0%)	148 (11.9%)	1.42 (0.74–2.75)	0.293	1.75 (0.76–4.01)	0.190
**Duration of living**						
< 2 years	8 (0.6%)	15 (1.2%)	Reference		Reference	
2–5 years	100 (8.1%)	52 (4.2%)	0.28 (0.11–0.70)	**0.006**	0.25 (0.07–0.84)	**0.024**
> 5 years	223 (18.0%)	842 (67.9%)	2.01 (0.84–4.81)	0.115	1.64 (0.51–5.23)	0.405

*COR*-Crude odds ratio

Table 3 shows identified health problems associated with cooking fuel type. The variables ascertained to be significant in the binary logistic regression analysis were added in the adjusted models. The adjusted model revealed that people who had cough related problems were 2.06 times more likely to have solid fuel use compared to people who had no cough related problems (AOR = 1.41, 95% CI = 0.80 – 2.92, *p* = 0.030). Participants who had pressure in chest while breathing were 2.52 times more likely have solid fuel user compared to those who had no pressure in chest while breathing (AOR = 2.52, 95% CI = 1.15 – 5.53, *p* = 0.021). Again, people who had discomfort in eyes were 2.31 times more likely to have solid fuel use compared to those who had no discomfort in eyes (AOR = 2.31, 95% CI = 1.18 – 4.55, *p* = 0.015). The adjusted model also revealed that participants who had no diabetes and didn’t know whether they had diabetes were 2.40 times and 9.54 times more likely to have solid fuel use (AOR = 2.40, 95% CI = 1.45 – 3.10, *p* < 0.001, AOR = 9.54, 95% CI = 4.06 – 22.41, *p* < 0.001, respectively) in comparison with those who had diabetes. People who didn’t know that they had asthma were 3.69 times more likely to have solid fuel user compared to those who had asthma (AOR = 3.69, 95% CI = 1.36 – 10.01, *p* = 0.011). Finally, the participants who didn’t know whether they had allergies were 35% less likely to have solid fuel use compared to participants who had allergies (AOR = 0.35, 95% CI = 0.14 – 0.87, *p* = 0.024).

**Table 3:** Binary and multivariate logistic regression analysis of health problems associated with fuel type

**Variables**	**Clean fuel** n (%)	**Solid fuel** n (%)	**Unadjusted model**		**Adjusted model**	
			COR (95% CI)	*p*-value	AOR (95% CI)	*p*-value
**Sneeze**						
Yes	9 (0.7%)	63 (5.1%)	2.67 (1.31–5.42)	**0.007**	1.42 (0.43–4.64)	0.562
No	322 (26.0%)	846 (68.2%)	Reference		Reference	
**Cough**						
Yes	22 (1.8%)	116 (9.4%)	2.06 (1.28–3.30)	**0.003**	1.41 (0.80–2.92)	**0.030**
No	309 (24.9%)	793 (64.0%)	Reference		Reference	
**Problems in breathing**						
Yes	25 (2.0%)	51 (4.1%)	Reference		-------------------	
No	306 (24.7%)	858 (69.2%)	1.37 (0.84–2.25)	0.209		
**Pressure in chest while breathing**						
Yes	18 (1.5%)	119 (9.6%)	2.62 (1.57–4.37)	** < 0.001**	2.52 (1.15–5.53)	**0.021**
No	313 (25.2%)	790 (63.7%)	Reference		Reference	
**Itching or burning in eyes**						
Yes	29 (2.3%)	181 (14.6%)	2.59 (1.71–3.92)	** < 0.001**	0.85 (0.43–1.69)	0.645
No	302 (24.4%)	728 (58.7%)	Reference		Reference	
**Redness in eyes**						
Yes	20 (1.6%)	96 (7.7%)	1.84 (1.12–3.03)	**0.017**	0.96 (0.44–2.10)	0.923
No	311 (25.1%)	813 (65.6%)	Reference		Reference	
**Discomfort in eyes**						
Yes	26 (2.1%)	196 (15.8%)	3.26 (2.10–4.96)	**0.001**	2.31 (1.18–4.55)	**0.015**
No	305 (24.6%)	713 (57.5%)	Reference		Reference	
**Diabetes**						
Yes	65 (5.2%)	89 (7.2%)	Reference		Reference	
No	232 (18.7%)	610 (49.2%)	1.92 (1.35–2.74)	** < 0.001**	2.40 (1.45–3.10)	** < 0.001**
Don’t know	34 (2.7%)	210 (16.9%)	4.51 (2.78–7.31)	** < 0.001**	9.54 (4.06–22.41)	** < 0.001**
**Hypertension**						
Yes	75 (6.0%)	187 (15.1%)	Reference		Reference	
No	205 (16.5%)	493 (39.8%)	0.97 (0.71–1.32)	0.821	1.10 (0.69–1.70)	0.744
Don’t Know	51 (4.1%)	229 (18.5%)	1.80 (1.20–2.70)	**0.004**	1.10 (0.59–2.10)	0.758
**Asthma**						
Yes	21 (1.7%)	70 (5.6%)	Reference		Reference	
No	294 (23.7%)	729 (58.8%)	0.74 (0.45–1.23)	0.252	0.81 (0.40–1.66)	0.558
Don’t know	16 (1.3%)	110 (8.9%)	2.06 (1.01–4.22)	**0.048**	3.69 (1.36–10.01)	**0.011**
**Allergy**						
Yes	109 (8.8%)	295 (23.8%)	Reference		Reference	
No	195 (15.7%)	496 (40.0%)	0.94 (0.71–1.24)	0.658	0.84 (0.57–1.24)	0.379
Don’t know	27 (2.2%)	118 (9.5%)	1.62 (1.01–2.59)	**0.047**	0.35 (0.14–0.87)	**0.024**

**Fig. 3:**
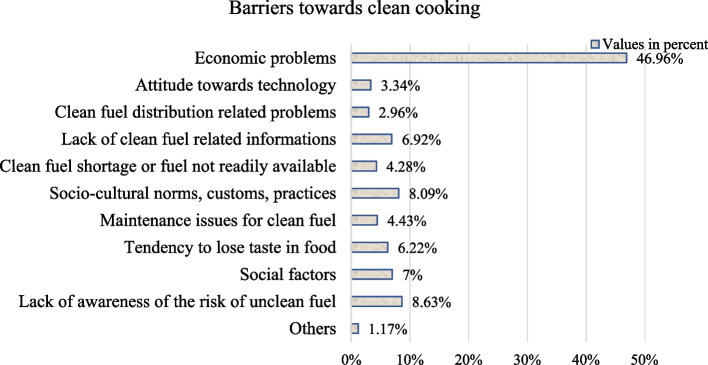
Barriers to clean cooking adoption

Figure 3 illustrates the barriers to clean cooking adoption. The data revealed that a higher percentage of participants (46.96%) cited economic problems as the primary reason for not using clean cooking fuel. Additionally, 8.63% of respondents identified a lack of awareness as a barrier, while 8.09% mentioned socio-cultural norms, customs, and practices. Furthermore, 6.22% of participants expressed concerns about losing the taste of food when cooking with clean fuel. Other barriers included social factors (7%), insufficient information about clean fuel (6.92%), maintenance issues with clean fuel (4.43%), a shortage of clean fuel or its unavailability (4.28%), attitudes towards technology (3.34%), problems related to the distribution of clean fuel (2.96%), and miscellaneous factors (1.17%), all contributing to obstacles in adopting clean cooking practices.

## Discussion

The use of solid fuel is a significant contributor to health problems in many low and middle-income countries, including Bangladesh. This study, to the best of our knowledge, is the first of its kind, covering four different divisions or regions in Bangladesh, aiming to investigate the association between health problems and cooking fuel type, along with barriers towards clean cooking. The findings revealed that approximately three-fourths of the participants used solid fuel for cooking. In the adjusted multivariate logistic model, the use of solid fuels was strongly associated with factors such as education, monthly family income, divisions/regions, and duration of living. Consequently, health problems such as cough, chest pressure while breathing, eye discomfort, diabetes, asthma, and allergies were strongly associated with solid fuel use. Economic problems were identified as the main barrier among others towards clean cooking.

Our study showed that 73.3% of rural households use solid fuel for cooking. While this finding is slightly more satisfactory than a previous study conducted in rural Bangladesh, which reported 94% of households using solid fuels for cooking [11], it still highlights a significant reliance on solid fuels. The government of Bangladesh has taken noteworthy initiatives for clean cookstoves to minimize solid fuel use [46]. Worldwide studies on the status of solid fuel use show diverse data, with around 80.5% of rural households in India using solid fuels [47], 95% in rural Pakistan [48], 95% in rural Myanmar [49], and 75% in rural China reported solid fuel use [50]. Factors associated with solid fuel use include economic problems, lack of awareness of the risks of solid fuels, and socio-demographic and socio-cultural influences [19].

A significant association was observed between illiteracy and the use of solid fuel, consistent with a previous cross-sectional study in Bangladesh that noted a higher prevalence of solid fuel use among individuals without formal education [39]. Similar patterns have been documented in other regions globally, such as in India [51] and Pakistan [52]. The likely causes contributing to this observation may include a lower awareness and a knowledge gap regarding the health risks associated with the use of solid fuel [18].

In the current study, a lower socioeconomic status (less than 15,000 BDT monthly) was significantly linked to the use of solid fuel. This finding aligns with a study in China, which reported that regions with low per capita household income are more reliant on solid fuel [50]. The prevalence of solid fuels in rural areas and the high prices of cleaner fuels compel households in rural Bangladesh to resort to cooking with solid fuel.

Our study revealed a significant association between cough, chest pressure while breathing, and eye discomfort with the use of solid fuel, consistent with similar findings in a study conducted in Bogura district, Bangladesh [31]. Comparable results have been reported in various parts of the world, including Nigeria [53], Uganda [54], China [55], and Nepal [12]. Moreover, diabetes, asthma, and allergies were also found to be significantly related to the use of solid fuel. Although a similar comprehensive study is lacking in Bangladesh, various global studies have addressed these issues. A cross-sectional study in China, for instance, established a strong connection between the use of household solid fuels and an elevated risk of diabetes [56]. Another study in India identified significant associations between asthma, allergies, and the use of solid fuel [57, 58].

Household members, particularly women and children, are highly exposed to household air pollutants such as respirable and fine particulate matter (PM2.5, PM10) and other gaseous substances released by solid fuels [26, 59]. These harmful air pollutants contribute to the development of asthma and allergy-related health problems in household members [60]. Additionally, PM pollution, particularly PM2.5, has a significant impact on blood glucose levels [61]. PM exposure elevates blood glucose levels through an increase in glucocorticoids [62]. Moreover, the majority of women who used solid cooking fuel experienced a negative impact on their blood pressure, a critical risk factor for diabetes mellitus [13]. Another study conducted in India reported that exposure to biomass fuel smoke significantly accelerates the prevalence of symptoms related to respiratory and eye problems [63]. Additionally, our findings indicate that economic problems are the main barriers to adopting clean cooking practices, consistent with results from a systematic literature review [19]. Furthermore, the tendency to lose food taste with clean fuels was identified as one of the significant barriers in Bangladesh.

Overall, this study provides valuable insights into the complex interplay of socio-demographic factors, health outcomes, and barriers influencing cooking fuel choices in rural Bangladesh. Addressing these factors is essential for promoting clean cooking practices, mitigating health risks, and contributing to sustainable development goals related to health and environmental sustainability.

### Strength and limitations of this study

The primary strength of this study lies in its extensive coverage of four different divisional regions, employing a large sample size to examine the association between health problems and cooking fuel type. The inclusion of an investigation into barriers toward clean cooking adds additional value, marking this study as the first of its kind in rural regions of Bangladesh. The findings from this study offer clear guidance to policymakers in formulating effective clean cooking plans. However, it is important to acknowledge the study’s limitations. The cross-sectional design impedes the establishment of causal relationships between solid fuel use and health outcomes. Additionally, the study’s focus on rural areas restricts the generalizability of findings to urban regions. Unmeasured factors could potentially influence the observed associations. Despite these limitations, the study provides valuable insights into the correlation between fuel type and health outcomes, as well as the barriers hindering the adoption of clean cooking practices in Bangladesh.

## Conclusion

In conclusion, this study underscores the substantial health risks associated with the persistent use of solid fuels for cooking in rural households in Bangladesh. Solid fuel use is linked to severe health consequences, including respiratory problems, eye ailments, diabetes, asthma, and allergies, compared to those using clean fuels. The findings emphasize the importance of considering cooking fuels as a significant factor in national-level policies and programs aimed at minimizing adverse health effects. Bangladesh’s ongoing efforts to achieve the SDGs, particularly the target of universal access to clean cooking energies, should prioritize addressing the implications of cooking fuel choices. Neglecting the development and implementation of technology for clean cooking compromises the attainment of other SDGs. This study contributes to policymaking by advocating for the adoption of clean fuels and raising awareness about the detrimental impacts of solid fuels. Collaborative efforts involving policymakers, local communities, and stakeholders, aimed at reducing barriers, can lead to meaningful improvements in clean cooking practices and overall well-being in rural areas. Future research, especially employing a qualitative approach, will further enhance our understanding of the current issues.

## Data Availability

The datasets used and/or analysed during the current study are available from the corresponding author on reasonable request.

## Abbreviations

SDGs: Sustainable Development Goals
WHO: World Health Organization
PM: Particulate Matter
SD: Standard Deviation
LPG: Liquified Petroleum Gas
AOR: Adjusted odds ratio
95% CI: 95% Confidence Interval
COR: Crude odds ratio
